# Tumor-Associated Neutrophils in Colorectal Cancer Development, Progression and Immunotherapy

**DOI:** 10.3390/cancers14194755

**Published:** 2022-09-29

**Authors:** Wei Zheng, Jingjing Wu, Yao Peng, Jing Sun, Pu Cheng, Qi Huang

**Affiliations:** 1 Department of Oncology, The Second Affiliated Hospital of Anhui Medical University, Hefei 230601, China; 2 Department of Pharmacy, The First Affiliated Hospital of Anhui Medical University, Hefei 230022, China; 3 Department of Gynecology, The Second Affiliated Hospital of Zhejiang University School of Medicine, Hangzhou 310052, China

**Keywords:** tumor-associated neutrophils, colorectal cancer, immune-checkpoint-inhibitor therapy

## Abstract

**Simple Summary:**

Tumor-associated neutrophils (TANs) may differentiate into different patterns under the stimulation of different factors, and they play a dual role in the occurrence and progression of tumors in direct or indirect ways. The existing immune checkpoint inhibitors (ICIs) are effective in a small number of microsatellite-instability-high (MSI-H) or mismatch-repair-deficient (dMMR) colorectal cancer (CRC) patients, but they are still not suitable for microsatellite-stability (MSS) CRC patients. As an important component of the tumor immune microenvironment, TANs may overturn the current situation of immunotherapy for CRC. This review systematically summarizes the key regulatory role of TANs in the carcinogenesis, proliferation and metastasis of CRC, the prognostic value of TANs for CRC patients and the new immunotherapy strategies based on TANs as a target, providing an important reference for TANs as new target for CRC immunotherapy.

**Abstract:**

The colorectal-cancer (CRC) incidence rate and mortality have remained high for several years. In recent years, immune-checkpoint-inhibitor (ICI) therapy has rapidly developed. However, it is only effective in a few CRC patients with microsatellite-instability-high (MSI-H) or mismatch-repair-deficient (dMMR) CRC. How to improve the efficiency of ICI therapy in CRC patients with microsatellite stability (MSS) remains a huge obstacle. Tumor-associated neutrophils (TANs), which are similar to macrophages, also have N_1_ and N_2_ phenotypes. They can be recruited and polarized through different cytokines or chemokines, and then play an antitumor or tumor-promoting role. In CRC, we find that the prognostic significance of TANs is still controversial. In this review, we describe the antitumor regulation of TANs, and their mechanism of promoting tumor progression by boosting the transformation of inflammation into tumors, facilitating tumor-cell proliferation, metastasis and angiogenesis. The targeting of TANs combined with ICIs may be a new treatment model for CRC. Relevant animal experiments have shown good responses, and clinical trials have also been carried out in succession. TANs, as “assistants” of ICI treatment, may become the key to the success of CRC immunotherapy, although no significant results have been obtained.

## 1. Introduction

Cancer may surpass cardiovascular diseases as a leading cause of death in many countries [1]. According to global cancer statistics in 2020, new cases and new deaths of colorectal cancer (CRC) account for 10.0% and 9.4% of all new cases and deaths worldwide, respectively [2]. The CRC incidence rate and mortality ranked among the top three in both men and women. With the improvement in the screening and treatment level, the incidence rate and mortality of CRC in developed countries have shown a decreasing trend. However, the incidence rate of CRC is rising rapidly in many developing countries, represented by China, with the changes in diet and lifestyle in recent decades [3,4]. CRC treatment mainly includes surgery, chemoradiotherapy and targeted therapy. Patients in different stages choose different treatment strategies according to the extent of the tumor invasion. Targeted therapies based on epidermal growth factor receptor (EGFR), vascular endothelial growth factor (VEGF), human epidermal growth factor receptor 2 (HER2), v-RAF murine sarcoma viral oncogene homolog B (BRAF) and other targets have been widely used, significantly improving the survival of CRC patients [5]. In recent years, immune checkpoint inhibitors (ICIs) based on programmed cell death protein 1 (PD-1), programmed cell death ligand 1 (PD-L1), and cytotoxic T lymphocyte-associated antigen-4 (CTLA-4), which can activate T cells to achieve antitumor effects, have achieved promising results. However, ICIs are not applicable for everyone. In CRC, less than 10% of patients with microsatellite-instability-high (MSI-H) or deficient-DNA-mismatch (dMMR) CRC showed a significant response to ICIs, while most microsatellite-stability (MSS)/proficient-mismatch-repair (pMMR) patients displayed poor efficacy [6]. Precision therapy based on operable targets is important to improve the survival of CRC patients.

The tumor immune microenvironment (TME) has been found to play a crucial role in tumor progression, including in CRC [7]. In the TME, the immune cells include T cells, natural killer cells, macrophages, neutrophils and so on. They all have different effects in antitumor immunity [8]. At present, there are relatively few studies on the neutrophil infiltration in the tumor microenvironment, and their functions have not been fully explained, which is still controversial. In human peripheral blood, neutrophils account for 50–70% of the circulating leukocytes. As short-lived cells, neutrophils play an indispensable role in both healthy and tumor tissues [9,10]. Similar to tumor-associated macrophages, neutrophils can differentiate into antitumor and protumor TANs under the chemotaxis of different factors, and they are also defined as N_1_ and N_2_, although it is unclear whether this classification is applicable to humans [11]. Interferon β (IFN-β) induces neutrophil polarization to an antitumor N_1_ phenotype [12], whereas transforming growth factor β (TGF-β) promotes the generation of protumor N_2_ neutrophils [11]. Interestingly, with the tumor progression, the N_1_ phenotype can turn into the N_2_ phenotype [13]. N_1_-TANs enhance the tumor cytotoxicity and attenuate immune suppression by producing tumor necrosis factor α (TNF-α), intercellular adhesion molecule-1 (ICAM-1), reactive oxygen species (ROS) and apoptosis-related factor (Fas), and by reducing the expression of arginase, while N_2_-TANs participate in tumor migration and metastasis through the expressions of arginase, matrix metalloproteinase 9 (MMP-9), VEGF and chemokines [11]. A number of researchers have reported that TANs play a crucial role in regulating the progress and prognosis of CRC, but the mechanism by which TANs regulate CRC remains poorly characterized. It is the purpose of this review to summarize the mechanism of TANs in the growth and prognosis in CRC, and to explore the possibility of targeting TAN therapy combined with ICIs as a new model in CRC clinical treatments.

## 2. Two-Faced Role of TANs in Tumor Progression

As the first line of defense against inflammation and infection, neutrophils are recruited from the vascular system to tissues via chemokines to play an anti-infection role. However, the dysregulation of neutrophil chemotaxis and activation may lead to a variety of diseases, including cancer [14]. The presence, recruitment and activation of TANs play a significant role in maintaining the TME and tumor progression.

A series of studies have revealed the possible antitumor mechanisms of TANs. Sunil Singhal demonstrated that the TAN subset from CD11b^+^CD15^high^CD10^−^CD16^low^ immature progenitors exhibited an antitumor function in the early stages of human cancer [15]. Neutrophils infiltrating cancer cells exert an antitumor function via the expressions of costimulatory receptors, including 4-1BBL, OX40L and CD86, thereby producing active T cells and secreting interferon γ (IFN-γ) [16]. Neutrophils are capable of directly killing cancer cells via the secretion of cytotoxic substances, such as ROS, nitric oxide (NO) and neutrophil elastase (NE) [17]. H_2_O_2_ secreted by neutrophils relies on the Ca^2+^ channel to kill cancer cells, which regulates the expression of transient receptor potential cation channel subfamily M member 2 (TRPM2) to inhibit cancer-cell proliferation [18]. Neutrophil-derived hepatocyte growth factor (HGF)-/mesenchymal–epithelial transition factor (MET)-dependent NO can promote the killing of cancer cells, which abates tumor growth and metastasis [19]. Tumor necrosis factor-related apoptosis-induced ligand (TRAIL) promotes cancer-cell death by binding to the TRAIL receptors on the cell surface, and it exhibits important antitumor activity. This mechanism has also been observed in chronic myeloid leukemia patients, inducing leukemia-cell apoptosis [20,21]. In addition to releasing cytotoxic substances, TANs can also release various chemokines and cytokines to stimulate the proliferation and activation of immune cells, such as T cells, NK cells and dendritic cells (DCs), thereby initiating antitumor immune responses [22]. CD8^+^ T cells can be recruited and activated by cytokines secreted by TANs, including the C-C motif chemokine ligand (CCL)-3, C-X-C motif chemokine ligand (CXCL)-10, TNF-α and interleukin (IL)-12 [23]. IFN-γ-stimulated TANs activate NK cells by releasing IL-18 [24], and TANs promote DC activation via the secretion of TNF-α [25]. Neutrophil-derived VEGF-_A165b_ mediates angiogenesis inhibition [26].

**Figure 1:**
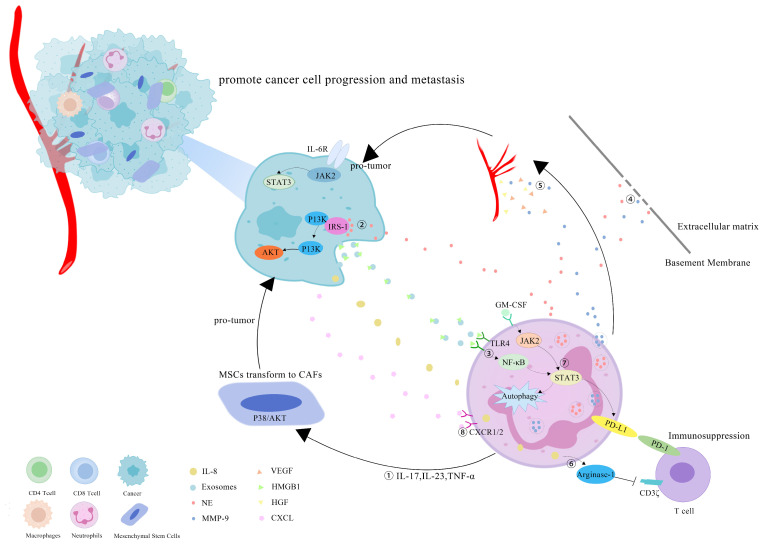
Mechanisms of TANs that promote tumor progression: (1) TANs secrete cytokines, such as IL-17, IL-23 and TNF-α, to induce MSCs to convert into CAFs, and to promote tumor-cell proliferation; (2) TANs secrete NE to bind intracellular IRS-1, releasing its inhibitory effect on the PI3K/Akt pathway, and promoting tumor proliferation; (3) cell-derived exosomes induce the autophagy and N2 polarization of neutrophils via HMGB1/TLR4/NF-κB signaling to promote cancer-cell proliferation and migration; (4) TANs secrete NE and MMP-9 to degrade the ECM and accelerate the tumor invasion; (5) TAN-derived VEGF, HGF and MMP9 promote the angiogenesis of tumor cells; (6) tumor-derived IL-8 induces neutrophils to secret arginase-1, resulting in arginase depletion and the establishment of an immunosuppressive TME; (7) GM-CSF activates TANs to express high levels of the immunosuppressive molecule PD-L1 through the JAK2/STAT3 signaling pathway; (8) neutrophils can be recruited by tumor cells through chemokines, such as the CXCL/CXCR1/2 signal axis.

However, more research has revealed that TANs may promote tumor progression through cancer-cell proliferation, invasion, angiogenesis and immunosuppression (Figure 1). Studies have investigated that TANs can induce mesenchymal stem cells (MSCs) to transform into tumor-related fibroblasts (CAF) by secreting IL-17, IL-23 and TNF-α, activating the protein kinase B/p38 (Akt/p38) pathway and ultimately promoting the proliferation and metastases of tumor cells [27]. IL-17 can also promote cancer-cell proliferation by activating the Janus kinase 2/signal transducers and activators of the transcription (JAK2/STAT3) pathway [28]. Moreover, neutrophils can be polarized into the N2 phenotype by tumor cells to promote the proliferation and migration of tumor cells. Tumor-cell-derived exosomes transport high-mobility group box-1 (HMGB1) to interact with Toll-like receptor 4 (TLR4) and activate the neutrophil nuclear factor kappa-B (NF-κB) pathway [29]. The tumorigenic mechanism of TANs also includes the reduction in the antitumor response of CD8^+^ T cells by the secretion of arginase-1, and the binding of TAN-derived NE to insulin receptor substrate-1 (IRS-1), both of which lead to cell proliferation [30,31,32]. TANs accelerate local tumor invasion by secreting MMP9 and NE to modify and degrade the extracellular matrix (ECM) [33]. HGF also contributes to local tumor invasion through the focal adhesion kinase (FAK)/paxillin signaling pathways [34]. Neutrophils have a unique ability to release chromatin reticulum, and namely, neutrophil extracellular traps (NETs). NETs can help circulating tumor cells enter the vascular system, promote their intravascular flow at the distal site and finally boost the invasion and metastases of tumor cells [35]. It has been shown that granulocyte macrophage colony-stimulating factor (GM-CSF), IL-5 and tumor-derived protease cathepsin C (CTSC) are all correlated with neutrophil recruitment and activation and promote lung metastases [36,37]. TAN-derived VEGF, HGF and MMP9 also make cancer cells more aggressive and facilitate angiogenesis [38]. Research conducted by Ting-ting Wang clarified that the JAK2/STAT3 signaling pathway is related to neutrophils in tumor immunosuppression, and it was shown that TANs were activated by GM-CSF and the induced high-level expression of the immunosuppressive molecule PD-L1 by the activation of the JAK2/STAT3 signaling pathway [39]. Tumor-derived IL-8 induces neutrophils to secret arginase-1, resulting in arginase depletion and the establishment of an immunosuppressive TME [40]. Moreover, chemokines produced by tumor cells, such as the CXCL1,2,5,8/CXCR1/2 signaling axis, can promote neutrophil recruitment, forming positive feedback with the tumor-promoting effect of TANs [41,42]. Chemokine receptors, such as CCR2 and CCR5, have also been implicated in neutrophil mobilization, recruitment and tissue infiltration [43,44].

## 3. The Prognosis Value of TANs in CRC

TANs, as an important prognosis factor, have been mentioned repeatedly in multiple solid tumors, including CRC, but the correlation between tumor-infiltrating neutrophils and the prognosis of CRC is still controversial. Several reports have shown that TANs are associated with a better prognosis. For example, Berry and his colleagues manually counted neutrophils based on the cell morphology and confirmed that high levels of TANs were associated with an improved overall survival in patients with stage II CRC [45]. Recently, Galdiero et al. analyzed 271 patients with stages I–IV CRC, and they described that the patients with higher TANs appeared to have better outcomes and better responses to 5-FU-based chemotherapy [46]. However, contrary to the previous studies, some reports tend to identify TANs as an unfavorable prognosis marker for CRC. It has been reported that the increase in TANs in CRC may lead to a worse prognosis and tumor progression [47,48,49]. Rottmann et al. found that high TANs conferred poorer prognosis in 348 patients with CRC, regardless of the MMR status [50]. Hu et al. identified CEACAM8 as a marker for detecting TANs in CRC, and they demonstrated that high-CEACAM8^+^ TANs were correlated with a worse DFS [51]. Several studies have proposed that TANs may have no significant impact on the prognoses of patients by analyzing the infiltrating immune cells of CRC [52,53]. The studies with contradictory results are summarized in Table 1.

One of the reasons why TANs may have different prognostic significances in different reports may be that there is no unified standard for the count of neutrophils in CRC tissues. The human immune system is very different from that of mice. In humans, neutrophils are defined as CD66b^+^CD33^+^CD15^+^CD14^−^, while in mice, they are frequently defined as CD11b^+^Ly6C^int^Ly6G^high^. The surface phenotype of neutrophils changes with the neutrophil differentiation. CD14, CD31 and CD64 are only expressed on activated neutrophils [71]. As a marker of neutrophils, CD66b is often used in the recognition and detection of neutrophils, but eosinophils may also express this marker, which may affect the accuracy of the results [72], and small-sample studies may lead to more inaccurate results. Moreover, using myeloperoxidase (MPO) or CEACAM8 to label neutrophils may yield a prognostic trend that is opposite to that of CD66b [46,51,73]. Another important reason is that the status of TANs is related to the tumor location, tumor stage and even the patient’s gender. For example, TANs usually exert an antitumor effect in early-stage patients. An analysis involving more than 1000 patients also confirmed that the accumulation of TANs in tumor stroma was correlated with a favorable prognosis [74]. More interestingly, some significant differences were observed only in female patients, but not in men [74]. Ignoring these factors may lead to different results. In order to accurately describe heterogeneous “neutrophil” populations, it is necessary to explore more accurate detection technology, such as combining more markers that may be expressed in neutrophils, and carefully stratifying patients, which will help to determine the prognostic significance of TANs.

## 4. Antitumor Effect of TANs in CRC

TANs may exhibit an antitumor effect in the early stages of tumor development. In an early-stage tumor, CD62L^low^CD54^high^ neutrophils facilitate T-cell growth and IFN-γ release [16]. The IFN signal activates the antitumor activity of the neutrophils, which express high levels of TNF-α, ICAM-1 and CCL3, and low levels of arginase-1 [75]. These have been verified in early-stage animal models of CRC. Neutrophils reduce the cancer-related inflammation caused by IL-17, and they inhibit the growth and progression of colon cancer by limiting the number and diversity of bacteria [76]. In addition, neutrophils interact with other immune cells to enhance the antitumor effect. For example, neutrophils were found to colocalize with CD8^+^ T cells in CRC. Neutrophils can enhance the responsiveness of CD8^+^ T cells to T-cell receptors, triggering and advancing the activation and proliferation of CD8^+^ T cells [59].

It is regrettable that only a few reports support the favorable role of TANs in CRC. More specific evidence to explain this viewpoint is lacking. In brief, only a few studies insist that TANs have the effect of inhibiting CRC growth, and mainly in the early stage of cancer. A more in-depth exploration of how the function of TANs changes with the stages of cancer would be helpful to understand the dual role of TANs in CRC, as well as in early diagnosis and treatment.

## 5. Tumor-Promoting Effect of TANs in CRC

### 5.1. TANs Are Associated with the Transformation of Inflammation into CRC

In the 19th century, the pathologist Rudolf Virchow first proposed the hypothesis of inflammation cancer and suggested that cancer originated from the site of inflammatory-cell infiltration [77]. Over the decades of research, the hypothesis between inflammation and cancer has been validated in several cancers, such as liver cancer, gastric cancer and CRC [78,79]. Inflammatory bowel disease (IBD) is characterized by intestinal idiopathic inflammatory diseases, including Crohn’s disease (CD) and ulcerative colitis (UC). Among them, UC patients are more likely to develop CRC [80]. The colitis-associated cancer (CAC) exhibits inflammatory-cell infiltration and the increased expressions of inflammatory cytokines (IL-1β, IL-6, TNF-α) and chemokines (CCL2 and CXCL1) [81].

**Figure 2:**
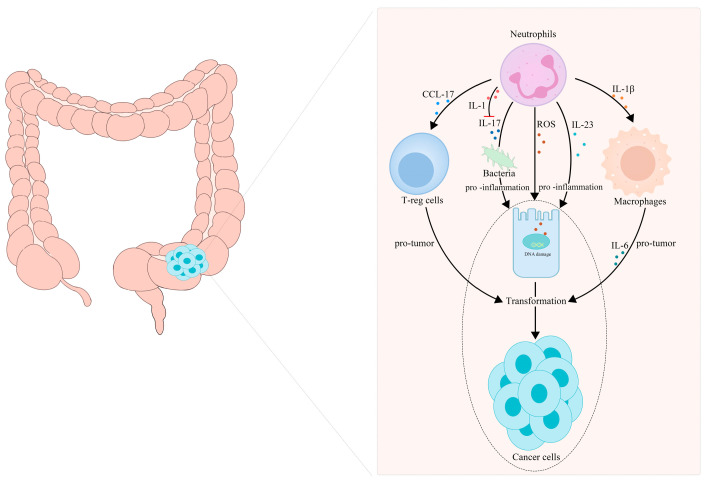
TANs participate in the transformation of inflammation into CRC. (1) TANs recruit T-reg cells and macrophages to participate in tumor progression by releasing CCL17 and IL-Iβ, respectively. (2) TANs release ROS, reactive nitrogen species and some enzymes to cause gene mutations and DNA damage, secrete IL-17 to maintain the diversity of the intestinal bacterial population and secrete interleukin, such as IL-23, to promote intestinal inflammation, which jointly promote the transformation of intestinal inflammation to tumor malignancy.

Neutrophil infiltration is a key event in chronic intestinal inflammation and CRC. Neutrophils, as important tumor-infiltrating immune cells, release a host of inflammatory factors, and they play a key role in CAC initiation and progression. Neutrophils infiltrating the intestine participate in the transformation from IBD to CAC by secreting IL-1β [82]. In 2015, Egle Kvedaraite and his colleagues proved, for the first time, that neutrophils infiltrating the colon tissue are the main source of IL-23 in IBD patients [83]. Neutrophils are considered to be the central effector cells of IBD. Apart from secreting IL-1β and IL-23, neutrophils can also produce ROS, reactive nitrogen species and some enzymes in IBD, leading to genetic mutations and DNA damage, and eventually transforming into cancer [84,85,86]. These substances secreted by neutrophils act at all stages of inflammation-cancer transformation and treatment. Azoxymethane/dextran sulfate sodium (AOM/DSS)-induced colitis has been used as a classic model for chronic-inflammation–cancer-transformation research. It is reported that there are a large number of CD11b- and Ly6G-labeled neutrophils in the colon tumor tissue formed in Apc^1638N/+^ mice repeatedly treated with AOM [87]. Infiltrated neutrophils can produce large amounts of IL-1β, which is critical for the development of CAC [82]. Chemokines secreted by TANs, such as CCL17, can inhibit the immune system and promote tumor progression by attracting regulatory T cells (T-reg) [88]. A statin hydroxamate synthesized by Tzu-Tang Wei prevented CAC in a mouse model by decreasing the infiltration of macrophages and neutrophils in the tumor-surrounding regions, and by reducing the inflammatory cytokines, chemokines and cyclin D1 in the tumor tissues [89]. It should be noted that neutrophil-specific IL-1 signaling can reduce intestinal inflammation and intestinal cancer invasion induced by inflammatory factors, such as IL-17, by limiting the number and diversity of bacteria [76,90]. A summary is presented in Figure 2.

The recruitment of neutrophils into the CRC niche involves a variety of chemotactic signals. The CXCL1/2/5-CXCR2 signal participates in promoting neutrophil recruitment to intestinal inflammatory mucosa and tumors. In an AOM/DSS-treated mouse model, it was observed that, during the transformation from enteritis to colorectal cancer, the infiltration of CXCR2-expressing neutrophils increased, and the chemokine CXCL1/2/5 accumulated in the inflamed colonic mucosa and tumors [41,91]. The loss of CXCR2 reduced the neutrophil infiltration from the circulatory system to the colitis mucosa and tumors [41]. Studies have shown that the inflammatory enzyme cyclooxygenase 2 (COX2), which is expressed in the inflamed colonic mucosa, can be induced by pathogenic bacteria from the intestine [92]. Moreover, the COX2- and COX2-derived prostate E2 (PGE2) was found to be significantly increased in the intestinal tracts of IBD patients [93]. In vivo and in vitro experiments have confirmed that the inflammatory mediator PGE2 markedly induced the expressions of the CXCR2 ligands CXCL1 and CXCL2 in intestinal mucosa and tumors, so as to attract CXCR2-expressing granulocytes to infiltrate the colonic mucosa and tumors [41,94]. The selective inhibition of COX2 by celecoxib can completely inhibit the expressions of CXCL1 and CXCL2 in intestinal tumors and mucosa and inhibit this chemotactic process [41]. Moreover, PGE2 is also reported to promote CRC tumorigenesis by stimulating the prostaglandin receptor EP2 in neutrophils to amplify the inflammation and shape the TME [95]. Spontaneous or therapy-induced apoptosis caused the accumulation of cell debris and induced the CD66b^high^CD11b^high^CD62L^low^ TAN phenotype in the CRC microenvironment. Apoptotic CRC cells release large amounts of IL-8, which can recruit neutrophils through the upregulated IL-8 reactive chemokine receptor CXCR2 on TANs in CRC tissues. In addition to IL-8, CXCL1 and CXCL5 are neutrophil chemoattractants, which are secreted by apoptotic CRC cells [96]. KRAS mutations are found in 40–50% of CRC cases. KRAS-mutant CRC cells were found to be able to induce neutrophil recruitment by upregulating IL-8 and transfer mutant KRAS to the neutrophils [97]. It has been reported that, in the neoplastic transformation during colorectal carcinogenesis, active neutrophil recruitment is often accompanied by the expression of calprotectin. The increased level of fecal calprotectin in CRC patients is most likely due to the neutrophil infiltration subsequent to the disruption of the mucosal integrity within the neoplastic colonic segment, resulting in the production of calprotectin, which is expressed in the cytoplasm of neutrophil granulocytes [98].

### 5.2. TANs Boost Proliferation, Migration and Chemoradiotherapy Resistance of CRC

Multiple studies have indicated that NETs play a role in tumor progression and metastases. The NETs released in the CRC microenvironment can profoundly stimulate the proliferation, invasion and migration of human and murine CRC cells under the irritation of tumor-cell-driven IL-8 [97,99]. NETs have also been shown to activate the TLR9-dependent signaling pathway in CRC cells to lead to cell growth, adhesion, migration and invasion [100]. The NET-affiliated protein carcinoembryonic Ag cell-adhesion molecule 1 (CEACAM1), which is responsible for decorating NETs, was proposed to participate in the adhesion, migration and metastases of colon carcinoma cells. CEACAM1 on NETs mediates the interaction between colon carcinoma cells and NETs, and it promotes colon-carcinoma-cell migration [101].

TANs have also been shown to produce soluble factors, such as cytokines and chemokines, which potentiate the cancer-cell survival and inhibit the response to therapy [17]. For example, TANs secrete anterior gradient-2 (AGR2) to promote CRC-cell migration via its receptor, CD98hc-xCT [102]. In vitro and mouse-model experiments showed that stimulation with CXCL2 increases the proliferation and adhesion of colon-cancer cells in a CXCR2-dependent manner [103]. It has been observed, in clinical treatment, that mild chemotherapy-induced neutropenia in multiple tumor species, including CRC, is often associated with an improved therapeutic response and prognosis [104,105,106,107]. Although this association was initially considered a coincidence, the similar trends in these studies are noteworthy because they indicate that the inhibition of TANs may improve the response to chemotherapy, independently of other confounders. Neutrophil-dependent chemoresistance is reflected in Dr. Nefedova’s team’s discovery that neutrophils promote multiple-myeloma-cell survival from doxorubicin [108]. Moreover, Shinde Jadhav et al. demonstrated the role for NETs as players in radio resistance in a mouse model of invasive bladder cancer [109]. However, up to now, no publications about the TAN involvement in the chemoradiotherapy resistance of CRC cells have been found.

In addition to tumor cells, TANs also have a certain impact on other cells in the TME. Germann et al. proposed that TANs significantly inhibited the T-cell activity in the TME. They reported that MMP-9 secreted by TANs converted the TGF-β precursor into an active form to exert immunosuppression and promote tumor progression in an inducible-colon-tumor mouse model. The public CRC gene expression datasets verified that the T-cell activity is lowest in human CRC with combined neutrophil infiltration and TGF-β activation [110]. In human CRC, neutrophils are found to colocalize with apoptotic tumor cells and macrophages. As mentioned above, apoptotic cancer cells attract neutrophils into tumors by releasing chemokines, such as IL-8, in which neutrophils can induce anti-inflammatory macrophage polarization. This interaction promotes the immunologically unfavorable tumor microenvironment, which may contribute to the tumor recurrence after chemoradiotherapy-induced apoptosis [96].

### 5.3. TANs Accelerate Liver Metastases of CRC

With the progress of treatment, the overall survival rate of patients with CRC has significantly increased. However, the 5-year survival rate of patients with distant metastases remains at only 19% [111]. The liver is one of the most common target organs for blood metastases of CRC. About 50% of CRC patients will develop liver metastases, and 25% of patients with CRC will still have liver metastases after operation [112]. With the in-depth study of the mechanism of the liver metastasis of CRC, more and more mechanisms and targets have been discovered and proposed, and the role of TANs in the liver metastases of CRC has come to light. The premetastatic niche is a frontier research direction. A key factor to promote tumor metastasis is to form a microenvironment that is conducive to tumor metastasis at a specific location, which is called the premetastatic niche [113]. As a fertile “soil”, the formation of the premetastatic niche is conducive to the colonization of metastatic tumor cells as proliferating “seeds”. Neutrophils contribute to each step of the metastasis cascade, including the formation of a premetastatic niche, the invasion of tumor cells into the endothelium and blood vessels and the extravasation of the distant-metastases tissue bed [114,115,116]. In CRC patients, the tissue inhibitor of the metalloproteinases 1 (TIMP-1) level is correlated with liver metastases. In mice, high systemic TIMP-1 levels induced neutrophil recruitment to the liver through the stromal-cell-derived factor-1 (SDF-1)/CXCR4 signaling pathway, triggering the formation of the liver premetastatic niche and increasing the liver susceptibility to metastases [117]. Owen J. Sansom’s team found that the epithelial Notch-1 signal could drive metastases through TGF-β-mediated neutrophil recruitment and infiltration through a genetically engineered mouse model of metastatic CRC [118,119]. SMAD family member 4 (SMAD4) is a downstream mediator of the TGF-β-signaling superfamily, which can play a tumor-suppressive role in colon carcinogenesis. TGF-β can induce SMAD4 inactivation in cancer cells, recruit CCR1^+^ neutrophils through the CCL15/CCR1 axis and mediate the progression and metastases of CRC [120,121,122]. The loss of SMAD4 was also found to recruit CXCR2^+^ neutrophils through the CXCL1/8-CXCR2 axis [123].

Besides facilitating the formation of a premetastatic niche, neutrophils promote the liver metastases of CRC by forming NETs [100]. In patients with metastatic CRC who tried radical hepatectomy, the formation of postoperative NETs was significantly correlated with *a* > 4-fold reduction in the DFS. Mouse neutrophil-derived NETs have been proven to trigger the release of HMGB1, and to promote the adhesion, proliferation, migration and invasion of tumor cells [100]. The elevated tumorous IL-8 expression triggered by NETs, in turn, activates neutrophils towards NET formation, thus forming a positive loop that optimizes CRC liver metastases [124]. The tumor microenvironment polarizes neutrophils. For example, fibroblast growth factor 2 (FGF2) expressed by metastasis-related neutrophils is significantly higher than that of primitive neutrophils. Therefore, Gordon-Weeks attempted to directly eliminate neutrophils, or deplete phenotypic neutrophils, with a FGF2-neutralizing antibody, which significantly inhibited the growth of liver metastatic colonies in mice, reduced the vascular density and branching and tended to normalize the blood vessels [125].

### 5.4. TANs Promote Angiogenesis in CRC

Angiogenesis plays an important role in tumor development and metastases, and angiogenesis inhibitors are an important clinical treatment [126]. Within the past few years, the combination of the antiangiogenesis drug Bevacizumab and chemotherapy has improved the prognoses of patients with CRC [127]. TANs have a powerful ability to promote the angiogenesis of CRC through multiple mechanisms. NE and MMP-9 secreted by TANs can degrade the basement membrane and ECM, trigger the release of bound VEGF-A and promote angiogenesis [17]. In addition, as a powerful angiogenic factor, HGF can function by directly acting on the cell-adhesion complexes, and by indirectly stimulating the production of IL-8 and VEGF [34]. FGF2, which is abundantly expressed in neutrophils, is also a strong angiogenic factor [125]. Vincent et al. discovered that the high expression of lysyl oxidase-like 4 (LOXL4) in CRC neutrophils is considered to be the key factor that leads to the resistance to antiangiogenic therapy, revealing a new mechanism of LOXL4 in neutrophil-mediated angiogenesis [128]. Bv8, known as prokineticin (Prok)1/2, has been proven to be significantly elevated in the plasma of patients with CRC. In a CRC mouse model, Bv8 was also strongly expressed in TANs. Bv8 induces endothelial-cell proliferation and angiogenesis by activating the VEGFR/protein kinase R (PKR) signal. Blocking neutrophil-derived Bv8 can improve the efficacy of anti-VEGF antibodies in the treatment of CRC [129].

## 6. TANs May Become a Potential Adjuvant Target for CRC Immunotherapy

In recent years, the rapid development of tumor immunotherapy has provided more options for the treatment of some malignant tumors. It has even revolutionized the treatment standards of some tumors, and it has become one of the most important and promising treatment methods, in addition to surgery, chemoradiotherapy and targeted therapy [130]. Tumor immunotherapy shows great potential in tumor treatment, which brings hope to tumor patients. Recent studies on immunotherapy primarily focus on activating the immune responses and removing tumor cells by activating immune cells. This immunotherapy can make the body produce a tumor-specific immune response through active or passive means to exert its function of inhibiting or killing tumors, and it can be divided into four categories: nonspecific immunostimulation, the immune checkpoint blockade, tumor-specific vaccines and adoptive immune-cell therapy [131]. In most solid tumors, the blockade of immune checkpoints, such as PD-1, PD-L1 and CTLA-4, is still the predominant form of tumor immunotherapy. In clinical treatment, ICIs have shown a significant effect on metastatic CRC patients with MSI-H/dMMR, but no exact efficiency was observed in MSS CRC patients, although the MSS CRC patients accounted for the majority of the total metastatic CRC patients [6]. Hence, MSS CRC is also regarded as a “cold” tumor by the industry. How to regulate the TME and convert a “cold” tumor to a “hot” tumor has become a huge bottleneck in the immunotherapy of advanced CRC. At present, the perspective of targeting protumor neutrophils has been proposed in several studies, which may become a key breakthrough point in the current immunotherapy dilemma of MSS CRC. 

TANs may be an important substantial barrier to the success of the current immunotherapy. Stimulated by TGF-β, TANs can produce NO or arginase-1, which inhibit the immune response and the infiltration of CD8^+^ T lymphocytes into the tumor environment [132]. TANs can also inhibit the T-cell proliferation by modulating PD-L1/PD-1 signaling [133]. Studies have found that PD-L1 is also expressed on neutrophils [134,135]. A large number of TANs expressed high levels of immunosuppressive PD-L1 in 105 patients with gastric cancer. PD-L1^+^ TANs have been proven to inhibit the proliferation and activation of T cells, as well as the adaptive response mediated by antitumor T cells in vitro. Therefore, PD-L1^+^ TANs promote the progression of disease and reduce the survival rate of gastric-cancer patients [39]. Studies have confirmed that targeted TAN therapy can improve the efficacy of a checkpoint blockade to a certain extent. CXCR1/2 inhibitors significantly inhibited the accumulation of TANs in tumor tissues and improved the efficacy of anti-PD-1 immunotherapy in mice [136]. In a CT26 mouse model of CRC, inhibiting phosphoinositide 3-kinases (PI3K)-δ/γ improved the efficacy of anti-PD-1 immunotherapy by targeting the immunosuppressive function of TANs [137]. Liver X receptor (LXR) can inhibit the neutrophil accumulation in tumors and their peripheries by regulating the expression of CXCL12. Mouse experiments have confirmed that using the LXR agonist RGX-104 can reduce the survival of TANs in mice and sensitize tumors to anti-PD-1 blocking antibodies [138]. In addition to animal studies, there are currently some clinical trials that combine targeted neutrophils with immunotherapy (Table 2; data were screened from ClinicalTrials.gov (accessed on 3 August 2022)). It has been proven that the preliminary results of combining cabozantinib and the PD-1/PD-L1 or CTLA-4 blockade in the treatment of tumors is promising. Cabozantinib is a pantyrosine kinase inhibitor that can simultaneously regulate the tumor-cell signaling pathways and neutrophil function [139]. Most clinical studies have not seen significant results yet; however, these studies provide a reference for TANs as a potential therapeutic target, and especially in improving or supplementing the existing immunotherapy.

Hence, the new approaches to cancer treatment may be to block the recruitment and polarization of neutrophils, or to selectively interfere with the tumor-promoting function of neutrophils. These strategies can be combined with ICIs to have a more positive therapeutic effect. However, so far, we still have a long way to go before we can fully understand and regulate the specific TAN function of cancer patients. If the protumor and antitumor effects of N_2_-TANs and N_1_-TANs are confirmed in humans, then the potential transformation from TAN2 to TAN1 may become a valuable strategy in cancer treatment. Theoretically, TGF-β blocking may be a potential therapeutic strategy due to TGF-β regulating the protumor and antitumor phenotypes of neutrophils, but TGF-β participates in a variety of physiological pathways, which cause significant side effects, which has led to the failure of a number of TGF-β-blocking trials [140]. So far, a TGF-β receptor II antibody (IMC-TR1, also known as LY3022859) exhibited significant inhibition on the growth and metastases of primary CRC, and it improved the antitumor efficacy of the cytotoxic agent cyclophosphamide. Tumor-bearing mice showed the enhanced apoptosis and necrosis of cancer cells. While no significant adverse reactions have been observed [141], high expressions of CXCR2 and CXCR1 were found in CRC cells [142]. Varney and colleagues reported that small-molecule antagonists suppress the activities of CXCR1 and CXCR2 and block the recruitment of neutrophils by the CXCL/CXCR1/2 axis, which results in the decreased neovascularization and increased apoptosis of malignant cells, and finally inhibits the liver metastases of colon cancer [142].

In the follow-up work, challenges and controversies in this research field still exist. How to target N_2_-TANs more precisely without disrupting the normal immune function of tumor patients is a critical issue. We believe that, with the progress of technology, and especially single-cell-profiling technology, the ability to classify heterogeneous neutrophil populations will be greatly enhanced, specific protein markers in N_2_-TANs will be pinpointed and N_1_-TANs will be retained to avoid neutropenia as the main side effect of neutrophil-targeted therapy. 

## 7. Conclusions and Outlook

ICI therapy for CRC has achieved remarkable results in MSI-H/dMMR CRC patients, but it has had little effect in the MSS type. Because MSS accounts for the majority of cases, it is urgent that we explore new methods to improve the efficacy of ICIs to improve the survival rates of patients. Continuous studies have clarified that the diversity and plasticity of TANs in the TME can escape therapeutic intervention [13,143]. Blocking the recruitment and polarization of neutrophils, or selectively interfering with the tumor-promoting function of neutrophils, may achieve antitumor therapy. Animal experiments and related clinical trials have also tried to combine targeted neutrophils and ICIs as a new treatment for CRC, and they have achieved promising initial results. Despite some side effects, the targeted therapy of TANs will still be a valuable strategy in the immunotherapy of CRC. In conclusion, the development of a tumor therapy that targets TANs may be a new era of immunotherapy for CRC.

## Figures and Tables

**Table 1:** Literature summary of prognostic role of TANs in CRC.

Prognosis	First Author	Year	Journal	Model	Keynote	Reference
Better	Chengzeng Yin	2022	Oncology Letters	Human	High density of CD66b^+^ TANs in the invasive margin significantly correlated with better prognoses for OS and DFS of patients with stages I–III CRC.	[54]
	Xiaowen Xu	2021	The Journal of Histochemistry and Cytochemistry	Human	Higher numbers of tumor-infiltrating CD66b^+^ neutrophils were significantly associated with both longer DFS and OS for CRC patients.	[55]
	Juha P Väyrynen	2020	Clinical Cancer Research	Human	Intraepithelial TANs and stromal TANs were significantly associated with better CSS and OS, respectively.	[56]
	Sofia Edin	2019	Scientific Reports	Human	Those highly infiltrated by CD66b^+^ cells had a significantly improved DSS.	[57]
	Lele Ye	2019	Frontiers in Immunology	Human	High-CD66b^+^ TANs were significantly related with better OS and DFS in CRC patients based on GEO and TCGA databases.	[58]
	Valeria Governa	2017	Clinical Cancer Research	Human	CD66b^+^ cell infiltration in CRC is significantly associated with increased OS.	[59]
	Maria L Wikberg	2017	Human Pathology	Human	Infiltration of CD66b^+^ cells in the tumor front indicated statistically favorable prognoses in patients with stages I–II colon cancer.	[60]
	Ryan S Berry	2017	PloS ONE	Human	High levels of TANs were associated with improved OS in patients with stage II CRC.	[45]
	Maria Rosaria Galdiero	2016	International Journal of Cancer	Human	CD66b was found to be a reliable marker to identify TANs in CRC tissues, whereas MPO also identified a subset of CD68^+^ macrophages. Higher TAN density was associated with better prognosis.	[46]
	Raoul A Droeser	2013	PloS One	Human	High MPO^+^ cell infiltration was significantly associated with better prognosis.	[61]
	Christian Hirt	2014	Oncoimmunology	Human	A high density of MPO^+^ infiltrating cells was significantly associated with increased 5-year OS.	[62]
	Hans Jorgen Nielsen	1999	Journal of Pathology	Human	High counts of neutrophils infiltrated in the peritumoral were significant predictors of good OS.	[63]
Worse	Yi Zhang	2022	Journal for Immunotherapy of Cancer	Human	The higher proportion of MPO^+^ cells in tumor-infiltrating stromal cells was significantly associated with worse prognosis.	[64]
	Katarzyna Jakubowska	2022	Oncology Letters	Human	Patients in the low-stroma TAN level group exhibited significantly longer 3- and 5-year DFS rates compared with those in patients in the high-stroma TAN level group.	[48]
	Bruce G Rottmann	2021	Journal of Clinical Pathology	Human	Patients with neutrophil-rich CRCs showed significantly poorer 5-year RFS compared with patients with neutrophil-intermediate or neutrophil-poor CRCs.	[50]
	Hao Su	2021	Journal of Cellular and Molecular Medicine	Human	Increased neutrophil infiltration in CRC was associated with a poorer prognosis based on data from GEO and TCGA databases.	[49]
	Xiang Hu	2019	Frontiers in Oncology	Human	CEACAM8 was used to detect tumor-infiltrating neutrophils within CRC. High-CEACAM8^+^ tumor-infiltrating neutrophils were associated with worse DFS.	[51]
	Yongfu Xiong	2018	Cancer Medicine	Human	Tumor-infiltrating neutrophils were significantly associated with poorer prognosis based on data from GEO and TCGA databases.	[65]
	Bing Zhu	2018	Cancer Medicine	Human	Increased CD66b^+^ TANs showed statistically unfavorable DFS and OS.	[66]
	Yihao Mao	2018	Cancer Management and Research	Human	High relative proportion of tumor-infiltrating neutrophils in colon cancer indicated poor OS based on data from GEO and TCGA databases.	[67]
	Hui-Lan Rao	2012	PloS One	Human	Increased intratumoral CD66b^+^ neutrophils were correlated with adverse OS.	[47]
No significance	Fang Jian	2022	International Immunopharmacology	Human	Tumor-infiltrating neutrophils had no significant effect on the OS of colon adenocarcinoma patients based on TIMER database (*p* = 0.406).	[52]
	Zigao Huang	2021	Frontiers in Oncology	Human	No significant association was found between tumor-infiltrating neutrophils and survival rates of patients with colon adenocarcinoma based on TIMER database (*p* = 0.789).	[53]
	Y Lin	2015	Clinical and Translational Oncology	Human	The number of tumor-infiltrating neutrophils (CD15^+^ neutrophils) did not significantly affect the overall survival.	[68]
	C H Richards	2012	British Journal of Cancer	Human	Peritumoral neutrophil infiltration was not significantly associated with CSS (*p* = 0.27).	[69]
	Do Trong Khanh	2011	Cancer Science	Human	The infiltration of neutrophils was not significant in predicting either RFS or OS in stages I–III CRCs (*p* = 0.410/*p* = 0.080, respectively).	[70]

OS: overall survival; DFS: disease-free survival; CSS: cancer-specific survival; DSS: disease-specific survival; RFS: recurrence-free survival.

**Table 2:** Ongoing clinical trials combining ICI blockades and agents related to neutrophil biology.

Ongoing Trials	Neutrophil Biology Targets	Agents	ICI Targets	ICIs	Cancer	First Posted	Phase	Status	Results
NCT02851004	STAT3	BBI-608	PD-1	Pembrolizumab	Metastatic CRC	2016	Phase Ib/II	Terminated	/
NCT03647839			PD-1	Nivolumab	MSS Metastatic CRC	2018	Phase II	Completed	Not available
NCT02983578		Danvatirsen/AZD9150	PD-L1	Durvalumab	dMMR CRC	2016	Phase II	Active, not recruiting	/
NCT03168139	CXCL12/CXCR4/CXCR7	NOX-A12/Olaptesed	PD-1	Pembrolizumab	Metastatic CRC	2017	Phase I/II	Completed	A total of 70% were still alive at 24 weeks, and 50% at 36 weeks. A total of 27% CRC patients achieved SD based on data from the 2018 ESMO Immuno-Oncology Congress.
NCT03473925	CXCR1/2	Navarixin	PD-1	Pembrolizumab	Advanced/Metastatic Solid Tumors (MSS CRC)	2018	Phase II	Completed	A total of 19 participants with MSS CRC were enrolled in a low-dose group (30 mg navarixin plus 200 mg pembrolizumab), and 21 participants in a high-dose group (100 mg navarixin plus 200 mg pembrolizumab). The median PFS was 1.8 months (95% CI, from 1.0 to 2.0) in the low-dose group, and 1.9 months (95% CI, from 1.6 to 2.0) in the high-dose group. The median OS was 6.5 months (95% CI, from 3.0 to 9.7) in the low-dose group, and 8.0 months (95% CI, from 5.7 to 14.4) in the high-dose group.
NCT04599140		SX-682	PD-1	Nivolumab	RAS-Mutated, MSS Metastatic CRC	2020	Phase Ib/II	Recruiting	/
NCT03184870	CCR2/5	BMS-813160	PD-1	Nivolumab	Advanced Solid Tumors (CRC)	2017	Phase Ib/II	Active, not recruiting	/
NCT03631407	CCR5	Vicriviroc	PD-1	Pembrolizumab	MSS Metastatic CRC	2018	Phase II	Completed	A total of 41 participants with MSS CRC were randomized to receive vicriviroc (low-dose: 150 mg; high-dose: 250 mg) in combination with pembrolizumab (200 mg). The ORRs of the two groups were both 5.0% (95% CI, from 0.1 to 24.9%). The median PFS was 2.1 months (95% CI, from 1.8 to 3.0) in the low-dose group, and 2.1 months (95% CI, from 1.6 to 3.9) in the high-dose group. The median OS was 4.6 months (95% CI, from 2.7 to 12.6) in the low-dose group, and 5.3 months (95% CI, from 3.2 to 8.0) in the high-dose group.
NCT04721301		Maraviroc	PD-1/CTLA-4	Nivolumab + Ipilimumab	Advanced Metastatic CRC	2021	Phase I	Active, not recruiting	/
NCT03274804			PD-1	Pembrolizumab	MSS Metastatic CRC	2017	Phase I	Completed	A total of 20 patients with MSS CRC received a pembrolizumab plus maraviroc treatment. After the core treatment period of 8 cycles, the DCR and ORR were both 5.3%. Median PFS was 9 weeks (95% CI, from 7.0 to 10.0), and median OS was 9 months (95% CI, from 6.0 to 20.0).
NCT03711058	PI3K	Copanlisib	PD-1	Nivolumab	MSS Metastatic CRC	2018	Phase I/II	Active, not recruiting	/
NCT02646748	PI3K-delta	INCB050465	PD-1	Pembrolizumab	Advanced Solid Tumors (CRC)	2016	Phase I	Completed	Not available
NCT05205330	PGE2	CR6086	PD-1	AGEN2034	pMMR-MSS Metastatic CRC	2022	Phase Ib/IIa	Recruiting	/
NCT03658772	PGE2-receptor/EP4	Grapiprant	PD-1	Pembrolizumab	MSS CRC	2018	Phase I	Active, not recruiting	/
NCT04432857		AN0025	PD-1	Pembrolizumab	Advanced Solid Tumors (MSS CRC)	2020	Phase Ib	Recruiting	/
NCT05205330		CR6086	PD-1	AGEN2034 /Balstilimab	pMMR-MSS Metastatic CRC	2022	Phase Ib/IIa	Recruiting	/
NCT04344795	EP2/EP4	TPST-1495	PD-1	Pembrolizumab	Solid Tumors (CRC)	2020	Phase Ia/Ib	Recruiting	/
NCT03026140	COX2	Celecoxib	PD-1/CTLA-4	Nivolumab/Ipilimumab	Early-Stage Colon Cancer	2017	Phase II	Recruiting	/
NCT03926338		Celecoxib	PD-L1	Toripalimab	CRC	2019	Phase I/II	Recruiting	/
NCT03638297	COX	aspirin	PD-1	BAT1306/pembrolizumab	MSI-H/dMMR Colorectal Cancer	2018	Phase II	Unknown	/
NCT02903914	Arginase	INCB001158/CB-1158	PD-1	Pembrolizumab	Advanced/Metastatic Solid Tumors (CRC)	2016	Phase I/II	Active, not recruiting	/
NCT03436563	TGF-βRII	M7824	PD-L1	M7824	CRC	2018	Phase Ib/II	Active, not recruiting	/
NCT02947165	TGF-β	NIS793	PD-1	PDR001	Advanced Malignancies (CRC)	2016	Phase I/Ib	Completed	Not available
NCT04429542	EGFR + TGF-β	BCA101	PD-1	Pembrolizumab	EGFR-driven Advanced Solid Tumors (CRC)	2020	Phase I	Recruiting	/
NCT04166383	TNF-α	VB-111	PD-1	Nivolumab	Metastatic CRC	2019	Phase II	Active, not recruiting	/
NCT04060342	CD11b	GB1275	PD-1	Pembrolizumab	MSS CRC	2019	Phase I	Terminated	/

SD: stable disease; ORR: objective-response rate; DCR: disease-control rate.
